# Transcription Factor E2F1 Exacerbates Papillary Thyroid Carcinoma Cell Growth and Invasion *via* Upregulation of LINC00152

**DOI:** 10.1155/2022/7081611

**Published:** 2022-05-10

**Authors:** Junjie Yang, Yong Ying, Xiangtai Zeng, Jiafeng Liu, Yang Xie, Zefu Deng, Zhiqiang Hu, Zanbin Li

**Affiliations:** Department of Thyroid and Hernia Surgery, The First Affiliated Hospital of Gannan Medical College, Ganzhou, China

## Abstract

**Background:**

Papillary thyroid carcinoma (PTC) is the most common thyroid neoplasm, whereas transcription factor E2F1 has been previously implicated in PTC progression. The current study sought to elucidate the underlying mechanism of E2F1 in PTC cell biological activities *via* regulation of long intergenic noncoding RNA 152 (LINC00152).

**Methods:**

Firstly, the expression patterns of LINC00152 and E2F1 in PTC were determined. Besides, TPC-1 and IHH-4 cells were adopted to carry out a series of experiments. Cell proliferation was detected by means of a cell counting kit-8 assay and colony formation assay, while cell migration and invasion abilities were assessed using a Transwell assay. Next, the interaction between E2F1 and LINC00152 was certified. Lastly, xenograft transplantation was carried out to validate the effects of E2F1 depletion on PTC.

**Results:**

Both LINC00152 and E2F1 were highly expressed in PTC cells. Knockdown of LINC00152 led to reduced cell activity, while LINC00152 overexpression brought about the opposing trends. Likewise, E2F1 knockdown quenched cell proliferation, migration, and invasion. However, the combination of E2F1 knockdown and LINC00152 overexpression resulted in augmented cell growth. In addition, E2F1 induced LINC00152 overexpression, which accelerated cell proliferation, migration, and invasion by activating the PI3K/AKT axis, whereas the administration of LY294002, the inhibitor of PI3K, led to reversal of the same. Finally, xenograft transplantation validated that E2F1 inhibition could suppress LY294002, thereby discouraging tumor growth.

**Conclusion:**

Our findings highlighted that E2F1 augmented PTC cell proliferation and invasion by upregulating LINC00152 and the PI3K/AKT axis. Our discovery provides therapeutic implications for PTC alleviation.

## 1. Introduction

As the most prevalent well-differentiated thyroid malignancy, papillary thyroid carcinoma (PTC) poses a great threat to patients owing to its increased likelihood of metastasis, significantly aggressive nature, and accompanying mortality [1]. Despite the tremendous advancements made in the treatment of PTC resulting in overall optimistic prognoses, the relapse rate of PTC covers almost one quarter during the follow-up period [2]. Further exacerbating its toll on society, PTC is frequently diagnosed in minors and young adult populations [3]. What is more, PTC has also been previously indicated as a potential cause of osteoporosis [4]. Exposure to iodine represents the most prominent pathogenesis of severe PTC [5]. In addition, a plethora of factors, including gender, age, lymph nodes, tumor volume, and extrathyroidal dissemination, are regarded as flagrant criteria for PTC prognosis assessment [6]. Surgical means and active surveillance are currently regarded the gold-standard for alleviating the plight of PTC; however, there is still a pressing need for more personalized interventions considering individuals' background and biological makeups [7, 8]. Towards this, we set out to explore the network of E2F1 in PTC with the interaction of the related genes.

E2Fs represent a class of transcription factors, implicated in a number of oncological processes, and further previously indicated as promising biomarkers of cancer prognosis [9]. Recent years have witnessed the emergence of E2Fs as a hot issue in the field of carcinoma research due to their ability to influence cancer cell activities, such as cell cycle and cell aggressiveness [10]. One such significant member of the E2F family, namely, E2F1, has been widely reported to function as a potent oncogene in human cancers including prostate cancer, lung cancer, and colorectal cancer by accelerating cancer cell invasion, dissemination, and metastasis and further predicting a poor prognosis [11–13]. What is noteworthy is that Yang and his colleagues shed light on the role of E2F1 in facilitating cell cycle, in addition to augmenting PTC tumor growth and invasion [14]. Another key focus of our study is long intergenic noncoding RNA 152 (LINC00152) which is overexpressed in PTC patients, wherein it aggravates cancer cell growth and colony formation *via* its interaction with upstream and downstream cytokines [15]. Furthermore, LINC00152 is also capable of exerting detrimental effects in a wide range of human malignancies by means of mediating cellular activities, enhancing lymph node metastasis and TNM stage, and also predicting high oncology recurrence and unsatisfactory overall survival rates [16, 17]. In lieu of these evidences, we hypothesized that the crosstalk between E2F1 and LINC00152 may be implicated in PTC progression and carried out a series of experiments to validate our hypothesis aiming to provide novel insight for PTC mitigation.

## 2. Materials and Methods

### 2.1. Ethics Statement

The current study was approved and supervised by the Ethics Committee of the First Affiliated Hospital of Gannan Medical College. Extensive efforts were undertaken to minimize both the number of experimental animals and their respective suffering.

### 2.2. Cell Cultivation and Treatment

PTC cell lines (TPC-1, BCPAP, and IHH-4) and thyroid epithelial cell line Nthyori3-1 (all purchased from ATCC, Manassas, Virginia, USA) were all cultured in 37°C RPMI-1640 medium (Gibco BRL, Carlsbad, CA, USA) containing 10% fetal bovine serum (FBS, GE Healthcare Life Sciences, Logan, UT, USA) with 5% CO_2_ in air.

LINC00152 cDNA was amplified using polymerase chain reaction (PCR) and then cloned into the pcDNA3.1 vector to construct pcDNA-LINC00152 (pc-LINC00152) plasmid. Short hairpin- (sh-) negative control (NC), sh-LINC00152-1, sh-LINC00152-2, sh-E2F1, sh-E2F1+pc-NC, and sh-E2F1+pc-LINC00152 plasmids were transiently transfected into TPC-1 cells; sh-NC and sh-LINC00152-1 plasmids were transfected into BCPAP cells, and sh-NC, sh-E2F1, pc-LINC00152, and pc-NC (empty plasmid) plasmids were transfected into IHH-4 cells. All the abovementioned plasmids were procured from Guangzhou RiboBio Co., Ltd (Guangzhou, Guangdong China), and the transient transfection was performed using the Lipofectamine 2000 reagent (Invitrogen, Carlsbad, CA, USA), in accordance with the manufacturer's instructions. After a 48-hour period of transfection, subsequent experimentation was carried out. To investigate the role of the PI3K/AKT axis in PTC cells, 10 *μ*M LY294002 (ApexBio, Houston, TX, USA), a PI3K inhibitor, was supplemented in IHH-4 cells for pretreatment for a duration of 2 h.

### 2.3. Cell Counting Kit-8 (CCK-8) Assay

Cells in each group (density of 2 × 10^3^ cells/100 *μ*L/well) were seeded into 96-well plates. Three duplicate wells were set at each time point at the 0 h, 24 h, 48 h, and 72 h time intervals. CCK-8 (Beyotime Biotechnology Co., Ltd, Shanghai, China) was added to cell-free medium to function as the NC. Next, these plates were cultivated in a 37°C incubator with 5% CO_2_ and then cultured in an incubator with 10 *μ*L CCK-8 added in corresponding wells at each time point for 4 h. Afterwards, optical density (OD) values at 450 nm were determined using a microplate reader.

### 2.4. Colony Formation Assay

Cells in each group were seeded into 6-well plates, at a density of 1 × 10^3^ cells/well. After the 2 to 3-week cultivation, plates were rinsed with phosphate buffer saline (PBS), fixed with 4% paraformaldehyde, and then stained using 0.1% crystal violet (Beyotime) for 5 min. Afterwards, the degree of colony formation was calculated and recorded by visual observation.

### 2.5. Transwell Assay

Firstly, the migration of cells was determined. Briefly, cells at the exponential phase were seeded into 6-well plates, with cell concentration was adjusted to 4 × 10^5^/mL after 48 h. Next, 500 *μ*L medium with 10% FBS was supplemented into the basolateral chamber of Transwell chamber, and 200 *μ*L cell suspension was added into the apical chamber. After 24 h of incubation at 37°C, the medium was discarded and chambers were placed in a clean 24-well plate with 500 *μ*L 4% paraformaldehyde added to each well. Subsequently, the chambers were fixed at room temperature for 30 min, stained with crystal violet for 60 min, and rinsed with PBS for 2-3 times. Afterwards, 5 images were captured from each well and analyzed.

Later, cell invasion was additionally documented as Matrigel (Franklin Lakes, NJ, USA) was spread on the Transwell chambers and then air-dried at room temperature. The subsequent steps were the same as the abovementioned details for migration assay.

### 2.6. RNA Immunoprecipitation (RIP) Assay

TPC-1 and IHH-4 cells were resuspended in nuclear isolation buffer, placed on ice for a minimum of 30 min, and mixed constantly. Next, the pellet nuclei were resuspended with wash buffer and 1× Protease inhibitors cocktail (Beijing Solarbio Science & Technology Co., Ltd., Beijing, China). Subsequently, the nuclei were used to shear the chromatin by means of high-power sonication. Then, 90% nuclei were cultured with 1 *μ*g E2F1 antibodies overnight and 40 *μ*L protein A/G beads (Thermo Fisher Scientific Inc., Waltham, MA, USA) at 4°C for 2 h *via* mild rotation, with the other 10% nuclei serving as the input. The pellet beads were centrifuged at 3000 rpm for 3 min and then rinsed thrice. Afterwards, LINC00152 expression patterns were detected by means of reverse transcription quantitative polymerase chain reaction (RT-qPCR).

### 2.7. Dual-Luciferase Reporter Gene Assay

The binding site between E2F1 and LINC00152 was verified using the JASPAR database (http://jaspar.genereg.net/). Wild-type (WT) and mutant-type (MUT) binding sites of the LINC00152 sequence were subcloned into the pGL3-basic vector (Promega, Madison, WI, USA) to establish LINC00152-WT and LINC00152-MUT plasmids, which were cotransfected into TPC-1 and IHH-4 cells with sh-E2F1 and sh-NC using the Lipofectamine™ 2000 reagent. After 48 h, luciferase activity was detected with the help of a fluorescence detector. All the aforementioned steps were repeated 3 times to obtain the mean value.

### 2.8. Xenograft Tumors in Nude Mice

A total of 12 nude mice (aged 6 weeks) (Hunan SLAC Laboratory Animal Co., Ltd., Changsha, Hunan, China) were randomly split into the sh-NC group and the sh-E2F1 group (with 6 mice in each group) to obtain the stably expressed TPC-1 cells in both groups. Next, 200 *μ*L cell suspension was subcutaneously injected into the right hind leg of mice. Tumor volume was measured using a vernier caliper every 7 days from the 7^th^ day until the 35^th^ day, when the mice were euthanized. Afterwards, the tumors were excised and weighed to collect tumor tissues for subsequent immunohistochemistry, RT-qPCR, and Western blot analysis.

### 2.9. Immunohistochemistry

The collected tumor tissues were fixed with 4% polyoxymethylene and made into paraffin-embedded sections, which were then cultured with the primary antibody anti-Ki67 (dilution ratio of 1 : 1500, BOSTER, Biological Technology Co., Ltd, Wuhan, Hubei, China) at 4°C overnight. The following day, the sections were cultivated with the corresponding secondary antibody labeled with horseradish peroxidase. The sections were subsequently assessed under a microscope after the visualization of Ki67 signal with the aid of 2,4-diaminobutyric acid. The sections were examined by two independent pathologists who were blinded to the experiment protocols. Ki67 staining was scored as the result of staining intensity multiplied percentage, with staining intensity graded four levels (no staining, 0; weak staining, 1; moderate staining, 2; and strong staining, 3), and the percentage of positive cells was classified into the following four grades: 0 (0%), 1 (1–25%), 2 (26–50%), 3 (51–75%), and 4 (76–100%).

### 2.10. RT-qPCR

TRIzol extraction kits (Invitrogen) were utilized to extract the total RNA content from tissues and cells. The primers (Table 1) used were synthesized by Takara Biotechnology Ltd. (Dalian, China). The obtained RNA was reverse-transcribed to cDNA using Rever Tra Ace Qpcr RT Master Mix kits (Toyobo Co., Ltd., Tokyo, Japan). Fluorescence qPCR was conducted using SYBR® Premix Ex TaqTM II kits, in accordance with the manufacturers' instructions. PCR conditions were as follows: predenaturation at 94°C for 4 min, 30 cycles of denaturation at 94°C for 30 seconds, annealing at 59°C, extension at 72°C for 1 min, and finally extension at 72°C for 5 minutes. The 2^-*ΔΔ*Ct^ method was adopted for expression calculation of genes, with glyceraldehyde-3-phosphate dehydrogenase (GAPDH) serving as the internal reference.

### 2.11. Western Blot Analysis

Total protein content was extracted from cells or tissues, followed by detection of protein concentration of each sample. Subsequently, the obtained proteins were transferred onto polyvinylidene fluoride membranes after separation with 10% sodium dodecyl sulfate polyacrylamide gel electrophoresis. Next, the membranes were sealed with 5% skim milk powder and then incubated with the following primary antibodies: E2F1 (ab112580, dilution ratio of 1 : 1000), phosphatidylinositol-3 kinase (PI3K) (ab32089, dilution ratio of 1 : 1000), p-PI3K (ab182651, dilution ratio of 1 : 1000), protein kinase B (AKT) (ab18785, 1/500), and p-Akt (ab38449, dilution ratio of 1 : 500) (all from Abcam Inc., Cambridge, MA, USA) at 4°C overnight. The following day, the membranes were cultured with secondary antibody immunoglobulin G antibody (ab97051, dilution ratio of 1 : 20000) (Abcam) at 37°C for 1 h through percussion. Afterwards, the membranes were immersed in an enhanced chemiluminescence reagent (Pierce, Waltham, MA, USA) for 1 min, followed by exposure, visualization and fixation in conditions void of light. GAPDH (ab9485, dilution ratio of 1 : 1000) (Abcam) was adopted as the internal reference. The Image J2x V2.1.4.7 software (Rawak Software, Inc. Dresden, Germany) was utilized for analysis of the Western blot images.

### 2.12. Statistical Analysis

Statistical analyses were performed using the SPSS 21.0 software (IBM Corp. Armonk, NY, USA). Normal distribution of data was ensured using the Kolmogorov-Smirnov test. Measurement data were shown in mean ± standard deviation. The *t*-test was utilized for analyzing comparisons between two groups, one-way and two-way analysis of variance (ANOVA) for comparing different groups, and Tukey's multiple comparisons test for pairwise comparisons following ANOVA. The *p* value was attained using a two-tailed test, and a value of *p* < 0.05 was regarded statistically significant.

## 3. Results

### 3.1. LINC00152 Promotes PTC Cell Growth and Invasion

**Figure 1:**
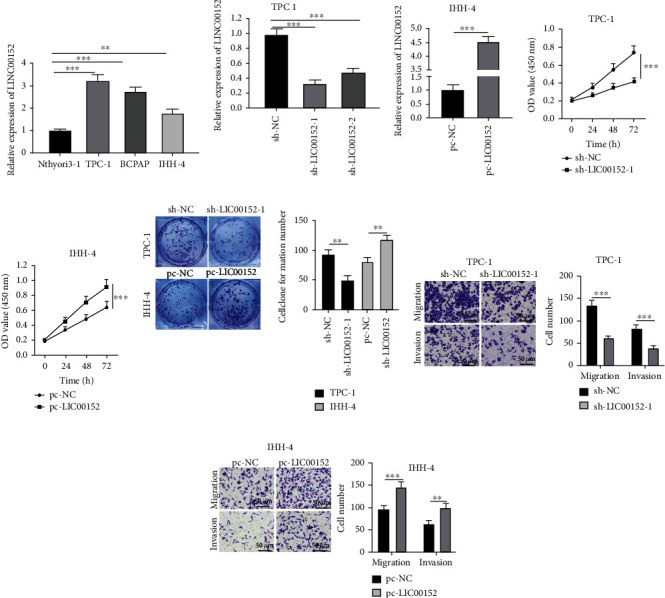
LINC00152 promotes PTC cell growth and invasion. (a) LINC00152 expression patterns in PTC cell lines measured by RT-qPCR. (b, c) Transfection efficiency of LINC00152 in (b) TPC-1 and (c) IHH-4 cells verified by RT-qPCR. (d, e) Proliferation of (d) TPC-1 and (e) IHH-4 cells detected by CCK-8. (f) Proliferation of TPC-1 and IHH-4 cells detected by colony formation assay. (g, h) Migration and invasion of (g) TPC-1 and (h) IHH-4 cells assessed via Transwell assay. Repetitions = 3. Data are expressed as mean ± standard deviation. The *t*-test was used for pairwise comparison. One-way ANOVA was used to determine statistical significance. Tukey's multiple comparisons test was applied for post hoc test. ^∗∗^*p* < 0.01; ^∗∗∗^*p* < 0.001.

Initial results of RT-qPCR demonstrated that LINC00152 expression levels were significantly higher in PTC cell lines (TPC-1, BCPAP, and IHH-4) compared to those in thyroid epithelial cell line Nthyori3-1, with the highest expression exhibited by the TPC-1 cells and the lowest expression by IHH-4 cells (all *p* < 0.01) (Figure 1(a)). To further elucidate the functioning mechanism of LINC00152 in PTC cells, LINC00152 (sh-LINC00152-1, the one with better knockdown efficiency, was selected for the further experiments) was knocked down in TPC-1 cells and simultaneously overexpressed in IHH-4 cells (all *p* < 0.001) (Figures 1(b) and 1(c)), followed by assessment of cell growth and invasion. The results of CCK-8 illustrated that LINC00152 knockdown led to reduced TPC-1 cell proliferation, while LINC00152 overexpression encouraged IHH-4 cell proliferation (all *p* < 0.001) (Figures 1(d) and 1(e)), and the same was confirmed by colony formation assay results (all *p* < 0.01) (Figure 1(f)). In addition, Transwell assay results demonstrated that migration and invasion of TPC-1 cell were both markedly reduced upon LINC00152 knockdown (all *p* < 0.001) (Figure 1(g)), whereas LINC00152 overexpression resulted in significant increases in migration (*p* < 0.001) and invasion (*p* < 0.01) of IHH-4 cells (Figure 1(h)). Additionally, LINC00152 was knocked down in BCPAP cells, and similar trends were documented (Supplementary Figure 1). Together, these findings indicated that LINC00152 knockdown could inhibit PTC cell growth and invasion.

### 3.2. E2F1 Induces the Upregulation of LINC00152 Expression

**Figure 2:**
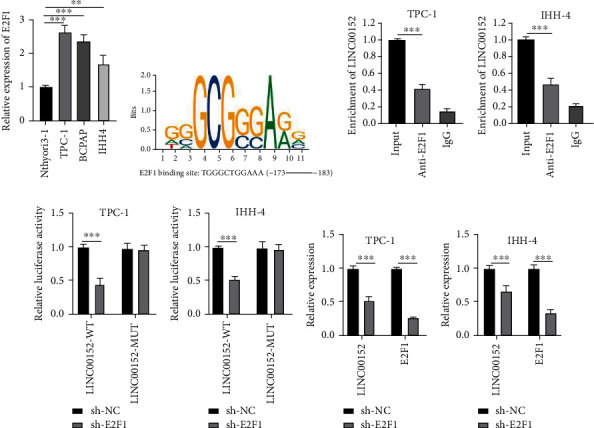
E2F1 induces the upregulation of LINC00152 expression. (a) E2F1 expression patterns in PTC cell lines measured by RT-qPCR. (b) Binding site between E2F1 and LINC00152 promoter predicted by JASPAR. (c, d) The interaction between E2F1 and LINC00152 in (c) TPC-1 and (d) IHH-4 cells verified by RIP. (e, f) The interaction between E2F1 and LINC00152 in (e) TPC-1 and (f) IHH-4 cells detected by dual-luciferase reporter gene assay. (g, h) Expression patterns of E2F1 and LINC00152 in (g) TPC-1 and (h) IHH-4 cells after silencing E2F1 assessed by RT-qPCR. Repetitions = 3. Data are expressed as mean ± standard deviation. The *t*-test was used for pairwise comparison. One-way ANOVA was used to determine statistical significance. Tukey's multiple comparisons test was applied for post hoc test. ^∗∗^*p* < 0.01; ^∗∗∗^*p* < 0.001.

Previously published literature suggests that E2F1 is capable of augmenting PTC cell proliferation and invasion by activating lncRNA RGMB-AS1 [18]. To further probe the role of E2F1 in PTC, we detected E2F1 expression patterns in PTC cells. As shown in Figure 2(a), E2F1 was highly expressed in TPC-1, BCPAP, and IHH-4 cells relative to Nthyori3-1 cells, with the highest expression exhibited by TPC-1 cells and the lowest expression by IHH-4 cells (all *p* < 0.01). The potential binding site between E2F1 and LINC00152 promoter was predicted using the JASPAR online webserver (http://jaspar.genereg.net) (Figure 2(b)). Subsequent results of RIP assay validated that E2F1 could directly bind to LINC00152 promoter in TPC-1 and IHH-4 cells (all *p* < 0.001) (Figures 2(c) and 2(d)). Moreover, dual-luciferase reporter gene assay results illustrated that E2F1 knockdown in TPC-1 and IHH-4 cells led to inhibition of LINC00152-WT luciferase activity (all *p* < 0.001) (Figures 2(e) and 2(f)). Meanwhile, both LINC00152 and E2F1 expression levels in the cells were both decreased after knockdown of E2F1 expression in TPC-1 and IHH-4 cells (all *p* < 0.001) (Figures 2(g) and 2(h)). Collectively, these findings indicated that E2F1 induced the upregulation of LINC00152 expression.

### 3.3. LINC00152 Overexpression Diminishes the Repressive Role of E2F1 Depletion on PTC Cell Growth and Invasion

**Figure 3:**
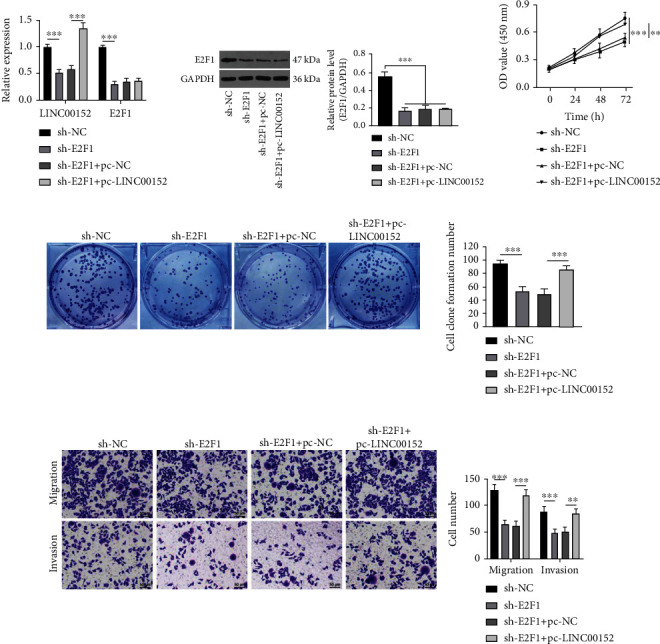
LINC00152 overexpression diminishes the repressive role of E2F1 knockdown on PTC cell growth and invasion. (a) Expression patterns of E2F1 and LINC00152 in TPC-1 cells assessed via RT-qPCR. (b) E2F1 expression patterns in TPC-1 cells calculated by Western blot analysis. (c, d) Cell proliferation detected by (c) CCK-8 and (d) colony formation assay. (e) Cell migration and invasion measured through Transwell assay. Repetitions = 3. Data are expressed as mean ± standard deviation. One-way ANOVA was used to determine statistical significance. Tukey's multiple comparisons test was applied for post hoc test. ^∗∗^*p* < 0.01; ^∗∗∗^*p* < 0.001.

To further investigate the effect of E2F1 on PTC cell growth and invasion, we knocked down E2F1 expression in TPC-1 cells and carried out functional rescue experiments in sh-E2F1+pc-NC and sh-E2F1+pc-LINC00152 groups. Subsequent results illustrated that the expressions of LINC00152 and E2F1 were both dramatically reduced after knockdown of E2F1. Compared with the sh-E2F1+pc-NC group, the sh-E2F1+pc-LINC00152 presented with unaltered expression of E2F1 and promoted LINC00152 expression levels (all *p* < 0.001) (Figures 3(a) and 3(b)). Moreover, E2F1 depletion led to significant attenuation of cell proliferation, migration, and invasion abilities (all *p* < 0.001), while the simultaneous knockdown of E2F1 and overexpression of LINC00152 brought about strengthened cell proliferation (*p* < 0.01), migration (*p* < 0.001), and invasion (*p* < 0.01) (Figures 3(c)–3(e)), indicating that LINC00152 overexpression could annul the repressive effect of E2F1 depletion on PTC cell growth and invasion.

### 3.4. LINC00152 Regulates PTC Cell Growth and Invasion via the PI3K/AKT Axis

**Figure 4:**
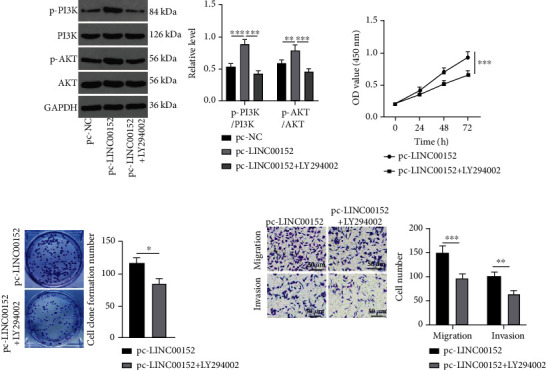
LINC00152 regulates PTC cell growth and invasion *via* the PI3K/AKT axis. Initially, 10 *μ*M LY294002 was added into IHH-4 cells to conduct pretreatment for 2 h. (a) Levels of the PI3K/AKT axis-related proteins (PI3K, p-PI3K, AKT, and p-AKT) verified through Western blot analysis. (b, c) Cell proliferation detected by (b) CCK-8 and (c) colony formation assay. (d) Cell migration and invasion measured through Transwell assay. Repetitions = 3. Data are expressed as mean ± standard deviation. The *t*-test was used for pairwise comparison. One-way ANOVA was used to determine statistical significance. Tukey's multiple comparisons test was applied for post hoc test. ^∗^*p* < 0.05; ^∗∗^*p* < 0.01; ^∗∗∗^*p* < 0.001.

To further elucidate whether LINC00152 could modulate PTC cell growth and invasion by activating the PI3K/AKT axis, we carried out a functional rescue assay as pc-LINC00152+LY294002 was administrated in IHH-4 cells. Subsequent results demonstrated that the PI3K/AKT axis was activated following overexpression of LINC00152, as evidenced by increased levels of p-PI3K/PI3K and p-AKT/AKT, while the administration of LY294002, an inhibitor of PI3K, led to the opposite trends (all *p* < 0.01) (Figure 4(a)). In addition, cell proliferation (*p* < 0.001), migration (*p* < 0.001), and invasion (*p* < 0.01) were all attenuated by LY294002 (Figures 4(b)–4(d)). Together, these findings indicated that LINC00152 could regulate PTC cell growth and invasion *via* the PI3K/AKT axis.

### 3.5. E2F1 Knockout Inhibits PTC Tumor Growth *In Vivo*

**Figure 5:**
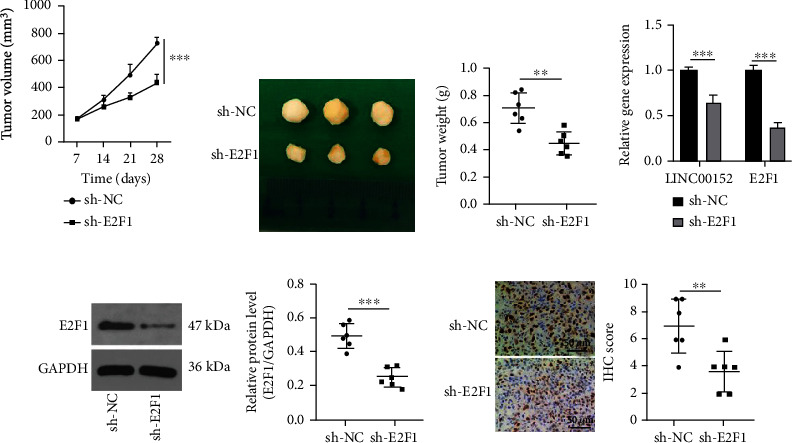
E2F1 knockout inhibits PTC tumor growth *in vivo*. (a) Tumor volume assessment. (b) Images of transplanted tumors in nude mice. (c) Weights of transplanted tumor in nude mice. (d) Expression patterns of LINC00152 and E2F1 mRNA detected by RT-qPCR. (e) Level of E2F1 protein verified through Western blot analysis. (f) Ki67 positive expression patterns measured by immunohistochemical staining. *N* = 6. Data are expressed as mean ± standard deviation. The *t*-test was used for pairwise comparison. ^∗∗^*p* < 0.01; ^∗∗∗^*p* < 0.001.

Lastly, E2F1 was underexpressed in nude mice, aiming to further explore the effect of E2F1 in PTC *in vivo*. Subsequent results illustrated that the depletion of E2F1 led to reduced transplanted tumor volume (*p* < 0.001) and weight (*p* < 0.01) *in vivo*, in addition to diminished LINC00152 and E2F1 expression levels (all *p* < 0.001), and decreased Ki67 positive expression (*p* < 0.01) (Figures 5(a)–5(f)). Altogether, these findings suggested that the inhibition of E2F1 reduced LINC00152 expression and consequently suppressed tumor growth *in vivo*.

## 4. Discussion

As the predominant form of thyroid cancer, PTC accounts for 80-85% of all thyroid cancer cases and is characterized by both favored survival rate and high aggressiveness and associated mortality [19]. Due to high risk of relapse and risky aftereffects, it is imperative to advance the search for active interventions against PTC [7]. Meanwhile, the hard-done work of our peers has shown the importance of lncRNAs in disease occurrence and development and their associations with the pathogenesis, diagnosis, and treatments of diseases [20]. Recent discoveries have also shed light on the ability of lncRNA to regulate cytokines at transcriptional, posttranscriptional, and epigenetic levels, to exert their influence on tumor progression [21]. One such lncRNA, namely, LINC00152, is known to function as a promotor in a wide range of tumors, including lung cancer, kidney cancer, gastric cancer, and gallbladder cancer, in line of its effects on cancer cell aggressiveness, invasion, and expansion [22]. Initial findings in our study indicated that LINC00152 exerted a promotive effect on PTC cell growth and invasion. LINC00152 expression is further highly expressed in PTC cells relative to healthy thyroid tissues [23], indicating the adverse effect of LINC00152 in PTC treatment. Moreover, LINC00152 has been previously implicated in the development of PTC due to its correlation with some other factors [15]. Accordingly, we sought to explore the possible gene that coordinates with LINC00152 to influence the fate of PTC.

The E2F family is widely implicated in cancer cell development and DNA spread, even in the context of thyroid malignancies [24]. Recent studies further documented the upregulation of E2F1, a transcription factor, in anaplastic TC cell lines, coupled with augmented cell cycle, underscoring the adverse effects of E2F1 in PTC [25]. A few studies have explored the role of E2F1 in PTC; there is a gray area in the specific crosstalk between E2F1 and other downstream genes involved in the biological activities of PTC. Interestingly, initial online prediction results in our study indicated that E2F1 could induce the upregulation of LINC00152. Moreover, as previously reported by Xia et al., LINC00152 can competitively combine with E2F1 to participate development of various cancers, such as prostate cancer, ovarian cancer, and PTC [26]. Thereafter, we carried out a series of functional rescue experiments where E2F1 was knocked down and LINC00152 was overexpressed in TPC-1 cells, in an effort to investigate the effect of E2F1 on PTC cell growth and invasion. Subsequent findings revealed that LINC00152 overexpression diminished the repressive effects of E2F1 knockdown on PTC cell growth and invasion. E2F1 is further renowned as a tumor promoter in numerous malignancies, due to its strong expressions in cancer tissues and cell lines, predicting undesirable prognoses, as well as enhancing cancer cell biological activities, metastasis, and metabolism [10]. Furthermore, prior evidence suggests that E2F1 is associated with frustrating overall survival and recurrence-free survival rates in individuals affected by PTC [27]. Further, in accordance with our data, the research carried out by Zhang et al. indicated that E2F1 exerted a promotive effect on PTC expansion, migration, and invasion by inducing the overexpression of a downstream gene [18]. In lieu of the abovementioned findings and evidence, it would plausible to suggest that the interaction between E2F1 and LINC00152 may be an impediment in PTC alleviation.

Another key focus of our research, the PI3K/AKT axis, is well established as a valuable biomarker of several human cancers owing to relation with molecular variances and intensified cellular activities [28]. Moreover, targeted interventions downregulating the PI3K/AKT axis represent a popular therapeutic regiment against advanced stage malignant tumors [29]. What is remarkable is that lncRNAs are also known to regulate certain pathways related to cancers, which underscores their utility in predicting cancer prognosis [30]. At that conjecture, we performed a functional rescue assay, where LINC00152 was overexpressed and the PI3K/AKT axis was downregulated in IHH-4 cells, aiming to figure out whether LINC00152 modulated PTC cell growth and invasion by activating the PI3K/AKT axis. The obtained findings unveiled that LINC00152 indeed enhanced PTC cell growth and invasion *via* the activation of the PI3K/AKT axis. Similarly, prior studies have shown that the PI3K/AKT axis aggrandizes PTC by enhancing cancer cell growth and spread [31]. Moreover, a recent study focusing on PTC documented that the PI3K/AKT axis was upregulated by an oncogene of PTC [32], which is in agreement with our findings. Besides, the study carried out by Xu et al. reported that LINC00152 was involved in the development of gastric cancer, liver cancer, and colon cancer, all by virtue of activating the PI3K/AKT axis [33]. In addition, we further validated the inhibitory effects of E2F1 knockout on PTC tumor growth *in vivo*, as evidenced by decreased Ki67 positive expression. The latter is particularly important as Ki67 positive expression is lauded as a critical indicator of PTC growth, since Ki67 was associated with tumor volume and thyroiditis and further associated with a high chance of relapse [34, 35]. Furthermore, a prior study highlighted that Ki67 expression was elevated in conjunction with E2F1 overexpression in cervical cancer, which reiterates the positive relationship between them [36]. All in all, the abovementioned findings indicate that knockout of E2F1 and LINC00152 is conducive to suppressing PTC.

In conclusion, our data sheds light on the mechanism wherein E2F1 augments PTC cell proliferation and invasion by upregulating LINC00152 and the PI3K/AKT axis. Our discoveries pave the way for novel therapeutic implications for PTC treatment. Currently, our study remains at the preclinical stage. Due to financial constraints, we were unable to uncover the upstream regulatory factors of LINC00152 by sequencing and other methods. In addition, there may be some other transcription factors involved in the role of LINC00152 in PTC. Nevertheless, we hope this study could be able to advance the search for effective therapeutic regimens against PTC. We shall strive to further explore the underlying mechanism in PTC and validate our findings at a clinical level in our future endeavors, hoping to contribute to PTC research.

## Figures and Tables

**Table 1:** Primers sequence of RT-qPCR.

Gene	Sequence
LINC00152	F: 5′-TTGATGGCTTGAACATTTGG-3′
	R: 5′-TCGTGATTTTCGGTGTCTGT-3′
E2F1	F: 5′-AGCGGCGCATCTATGACATC-3′
	R: 5′-GTCAACCCCTCAAGCCGTC-3′
GAPDH	F: 5′-GGTGGTCTCCTCTGACTTCAACA-3′
	R: 5′-GTTGCTGTAGCCAAATTCGTTGT-3′

Note: RT-qPCR: reverse transcription quantitative polymerase chain reaction; GAPDH: glyceraldehyde-3-phosphate dehydrogenase; F: forward; R: reverse.

## Data Availability

The data that support the findings of this study are available from the corresponding author upon reasonable request.

## Supplementary Materials

Supplementary Materials Supplementary Figure 1: the results suggested that LINC00152 knockdown could inhibit PTC cell growth and invasion. (A) LINC00152 knockdown efficiency in BCPAP cells measured by RT-qPCR. (B) BCPAP cell proliferation detected by CCK-8. (C) BCPAP cell proliferation detected by colony formation assay. (D) Migration and invasion of BCPAP cells assessed *via* Transwell assay. Repetitions = 3. Data are expressed as mean ± standard deviation. The *t*-test was used for pairwise comparison. ^∗∗^*p* < 0.01; ^∗∗∗^*p* < 0.001.
