# Supplementary material for: Transcription Factor E2F1 Exacerbates Papillary Thyroid Carcinoma Cell Growth and Invasion via Upregulation of LINC00152
Source: Anal Cell Pathol (Amst). 2022 May 10;2022:7081611. doi: 10.1155/2022/7081611 (PMC9113902; doi:10.1155/2022/7081611)
Supplement: Supplementary Materials — Supplementary Figure 1: the results suggested that LINC00152 knockdown could inhibit PTC cell growth and invasion. (A) LINC00152 knockdown efficiency in BCPAP cells measured by RT-qPCR. (B) BCPAP cell proliferation detected by CCK-8. (C) BCPAP cell proliferation detected by colony formation assay. (D) Migration and invasion of BCPAP cells assessed via Transwell assay. Repetitions = 3. Data are expressed as mean ± standard deviation. The t-test was used for pairwise comparison. ∗∗p < 0.01; ∗∗∗p < 0.001. [file 7081611.f1.docx]

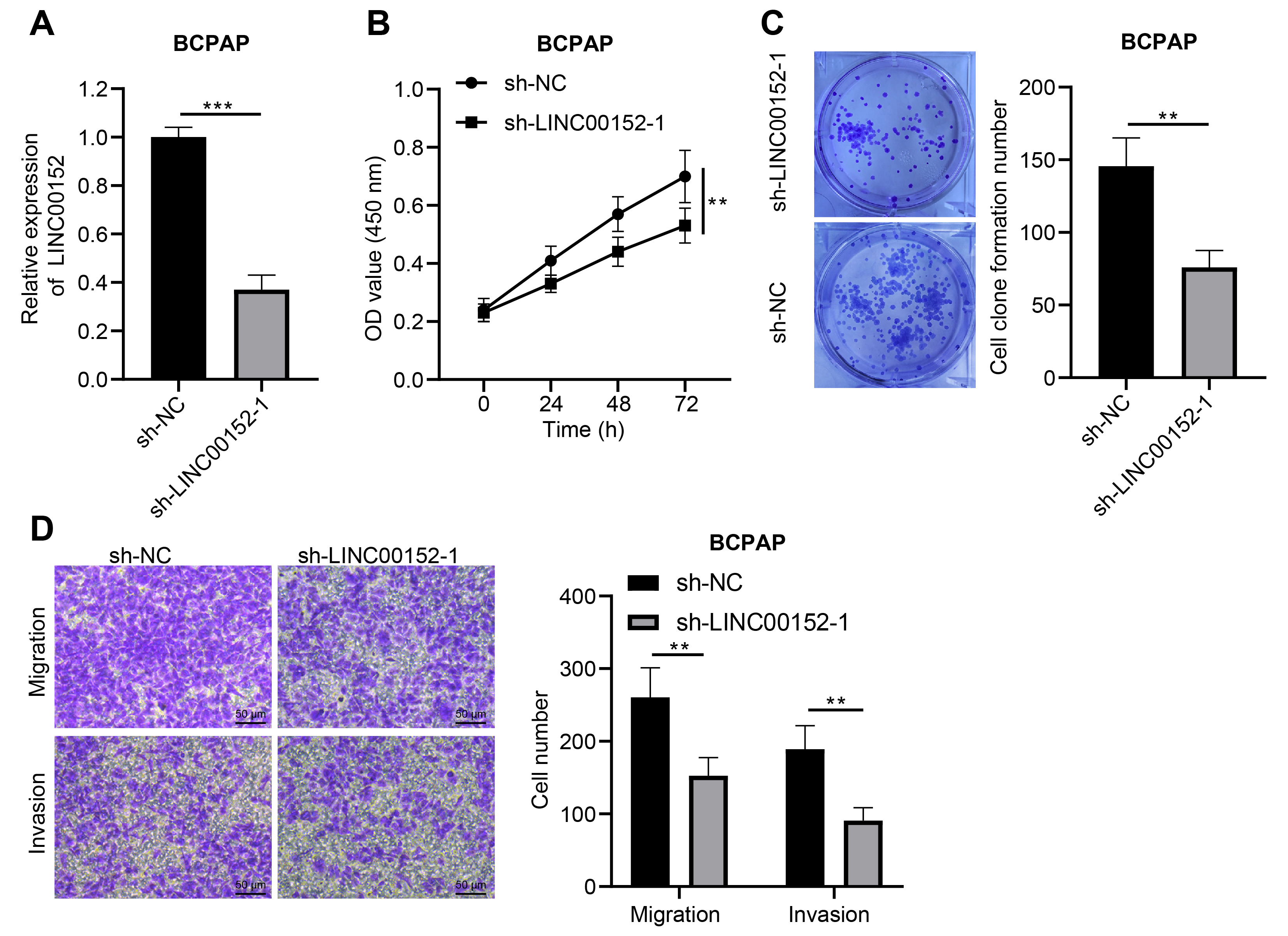


**Supplementary Figure 1** The results suggested that LINC00152 knockdown could inhibit PTC cell growth and invasion. A, LINC00152 knockdown efficiency in BCPAP cells measured by RT-qPCR. B, BCPAP cell proliferation detected by CCK-8. C, BCPAP cell proliferation detected by colony formation assay. D, migration and invasion of BCPAP cells assessed *via* Transwell assay. Repetitions = 3. Data are expressed as mean ± standard deviation. The t-test was used for pairwise comparison. ** *p*<0.01, *** *p*<0.001.
